# Return to Sport after Anatomic and Reverse Total Shoulder Arthroplasty in Elderly Patients: A Systematic Review and Meta-Analysis

**DOI:** 10.3390/jcm9051576

**Published:** 2020-05-22

**Authors:** Rocco Papalia, Mauro Ciuffreda, Erika Albo, Chiara De Andreis, Lorenzo Alirio Diaz Balzani, Anna Maria Alifano, Chiara Fossati, Andrea Macaluso, Riccardo Borzuola, Antonio De Vincentis, Vincenzo Denaro

**Affiliations:** 1 Department of Orthopaedic and Trauma Surgery, Campus Bio-Medico University of Rome, 00128 Rome, Italy; r.papalia@unicampus.it (R.P.); m.ciuffreda@unicampus.it (M.C.); c.deandreis@unicampus.it (C.D.A.); l.diaz@unicampus.it (L.A.D.B.); a.alifano@unicampus.it (A.M.A.); a.devincentis@unicampus.it (A.D.V.); v.denaro@unicampus.it (V.D.); 2 Department of Movement, Human and Health Sciences, University of Rome “Foro Italico”, 00135 Rome, Italy; chiara.fossati@uniroma4.it (C.F.); andrea.macaluso@uniroma4.it (A.M.); r.borzuola@studenti.uniroma4.it (R.B.)

**Keywords:** shoulder, arthroplasty, replacement, return to sport, elderly, systematic review, meta-analysis

## Abstract

The aim of this systematic review and meta-analysis was to evaluate the rate of return to sport in elderly patients who underwent anatomic (ATSA) and reverse (RTSA) total shoulder arthroplasty, to assess postoperative pain and functional outcomes and to give an overview of postoperative rehabilitation protocols. A systematic search in Pubmed-Medline, Cochrane Library, and Google Scholar was carried out to identify eligible randomized clinical trials, observational studies, or case series that evaluated the rate of return to sport after RTSA or ATSA. Six retrospective studies, five case series, and one prospective cohort study were included in this review. The overall rate of return to sport was 82% (95% CI 0.76–0.88, *p* < 0.01). Patients undergoing ATSA returned at a higher rate (90%) (95% CI 0.80–0.99, *p* < 0.01) compared to RTSA (77%) (95% CI 0.69–0.85, *p* < 0.01). Moreover, the results showed that patients returned to sport at the same or a higher level in 75% of cases. Swimming had the highest rate of return (84%), followed by fitness (77%), golf (77%), and tennis (69%). Thus, RTSA and ATSA are effective to guarantee a significative rate of return to sport in elderly patients. A slightly higher rate was found for the anatomic implant.

## 1. Introduction

Total shoulder arthroplasty (TSA) is the third most common replacement procedure after hip and knee arthroplasty and is considered the elective treatment for patients affected by advanced shoulder pathology with loss of function and severe pain [1]. Indeed, the main indications for shoulder replacement are primary and secondary glenohumeral osteoarthritis, osteonecrosis, and fractures of the proximal epiphysis of the humerus or its sequelae [1,2,3,4,5].

Three main different designs of shoulder prostheses allow surgeons to decide which is the best option for each specific case, trying also to meet patients’ health needs [6]. The reverse shoulder arthroplasty (RTSA) has a specific design that determines the medialization of the rotation center, permitting the recruitment of a large part of the deltoid muscle [7]. Thus, even if the rotator cuff is damaged, the arc of movement is preserved [7]. Conversely, the biomechanics of the anatomic total shoulder arthroplasty (ATSA) is based on rotator cuff integrity. The third implant option includes hemiarthroplasty (HHA) and humeral head resurfacing that differ from the above mentioned because they do not require glenoid replacement. Currently, these implants are more often indicated for the treatment of end-stage osteoarthritis as well as fractures of the humeral head in young patients [8]. As a consequence, the number of implanted HHAs has decreased in the last 15 years in favor of TSAs [1,9,10,11].

The constant increase of TSAs, in younger as well as older individuals, correlates with their good long-term outcomes and with the widening of surgical indications [1,2]. In this scenario, as life expectancy is increasing, older patients are also asking their surgeons for better outcomes in order to return to previous sports activities after surgery [3]. Return to sports activities at a preoperative level has widely been investigated for patients with hip and knee arthroplasties with satisfactory rates of return [4,5,6]. However, limited data exist regarding rates of return after TSA, even if this field is becoming of increasing interest. A recent multi-center international study, carried out by the American and European Society of Shoulder and Elbow Surgeons, stated that non-contact low-load activities with low risk of fall or collision were most frequently allowed by surgeons [7].

The TSA post-operative rehabilitation protocol plays an important role in active patients to achieve satisfactory post-operative clinical outcomes and range of motion (ROM) [8]. Despite the existence of a large number of studies about rehabilitation protocol after hip and knee replacement, there are still only a few papers focused on specific and standardized postoperative rehabilitation protocols for patients with TSA [9,10]. Current postoperative guidelines are usually based on the original protocol developed by Hughes and Neer [11]. This protocol contemplates different steps with a gradual mobilization of the arm, avoiding flexion and abduction movements until the fourth postoperative week, but it does not provide indications for rehabilitation finalized to the resumption of sports activities. Nevertheless, for these patients, a postoperative rehabilitation protocol with a specific exercise routine could be advisable [11,12,13].

The primary aim of this systematic review and meta-analysis is to evaluate the rate of return to sport in elderly patients after ATSA and RTSA. The secondary and tertiary endpoints focus on the assessment of postoperative pain and functional outcomes and to give an overview of current postoperative rehabilitation protocols.

## 2. Materials and Methods

The present systematic review and meta-analysis was performed according to the Preferred Reporting Items for Systematic reviews and Meta-Analyses (PRISMA) guidelines [14]. In this review, we included randomized clinical trials, observational studies, and case series, which evaluated the return to sport of cohorts with an average age greater than 65 years who underwent RTSA and ATSA.

### 2.1. Eligibility Criteria

According to the Oxford Centre of Evidence-Based Medicine, peer-reviewed studies of I to IV levels of evidence were considered for inclusion. Case reports, studies on animals, biomechanical reports, technical notes, letters to editors, instructional courses, cadaver or in vitro investigations, systematic reviews and meta-analyses, or studies without an abstract were excluded. According to the definition of the elderly by the WHO, the search was focused on papers reporting on return to sports of cohorts, with an average age greater than 65 years who underwent RTSA and ATSA. Only studies with a minimum of 10 patients and a minimum of one-year follow-up were included. Moreover, only articles in Italian, English, Spanish, and French were considered.

### 2.2. Study Outcomes

The primary outcome of the analysis was to establish the rate of return to sport of elderly patients after ATSA and RTSA. Among the included papers, the secondary endpoint was to assess the postoperative pain and functional outcomes, taking into account the reported standardized clinical scores. The tertiary endpoint of this systematic review was to give an overview of the proposed rehabilitation protocols.

### 2.3. Search Methods for Identification of Studies

A systematic literature search in Pubmed-Medline, the Cochrane Library and Google Scholar databases was carried out between September 2019 and April 2020. For Pubmed, the following search strategy was used: (((“Shoulder Joint”[Mesh] OR (“shoulder”[All Fields] AND “joint”[All Fields]) AND (“Arthroplasty”[Mesh] OR (“Arthroplasty”[All Fields]) OR (“Replacement”[All Fields])) AND (“Sports”[Mesh] OR (“return to sport” [All Fields] OR (“return” [All Fields] AND (“sport” [All Fields])) AND “Aged”[Mesh])). No time interval was set for publication date. Two independent reviewers (M.C. and C.D.A.) conducted the electronic search identifying the potentially relevant studies. Firstly, the retrieved articles were screened by title and, if relevant, by reading the abstract. After the exclusion of non-eligible studies, the full-text of the remaining articles was evaluated for eligibility. To minimize the risk of bias, the authors reviewed and discussed all the selected articles, the references, as well as the articles excluded from the study. If any disagreement between the reviewers was found, the senior investigator (R.P.) made the final decision. At the end of the process, further potentially missed studies were manually searched for among the reference lists of the included papers and the relevant systematic reviews.

### 2.4. Data Collection

All reviewers discussed the relevant items for data extraction before starting the process in order to avoid data omission. Data were independently extracted by two reviewers (M.C. and C.D.A.) and divergences were discussed with the third reviewer (R.P.) if necessary. All data related to primary, secondary and tertiary outcomes were summarized in standardized tables. Specifically, the following variables were recorded: authors, year of publication, type of study, level of evidence, number of participants, mean age, dominant or not-dominant limb, surgical approach, mean follow-up, complications, patients returned to sport and type of activity, secondary outcome measures, and rehabilitative protocols. Among the outcomes, we analyzed functional outcomes and severity of pain.

### 2.5. Risk of Bias Assessment

The quality of the included studies was independently evaluated by two reviewers (C.D.A. and M.C.) using the Methodological Index for Non-randomized Studies (MINORS) score [15]. The following domains were assessed: a clearly stated purpose, inclusion of consecutive subjects, prospective data collection, endpoints appropriate to the purpose of the study, unbiased assessment of the study endpoints, follow-up period appropriate for the study, loss to follow-up of less than 5%, prospective calculation of the study size, adequate control group, contemporary group, baseline group equivalence, and adequate statistical analysis. The last four items are specific to comparative studies. Each item was scored from 0 to 2 points, with a global ideal score of 16 points for non-comparative studies and 24 points for comparative studies.

### 2.6. Statistical Analysis

Meta-analysis was performed to determine the overall proportion of subjects returning to sport and the functional and pain level after shoulder arthroplasty across all the retrieved studies. Raw, i.e., untransformed, proportions and means were used to report the pooled proportions and means that were obtained with the inverse variance method. Heterogeneity was evaluated using Q statistic, expressed as the p value for the χ^2^ test under the null hypothesis that the between-study variance (τ^2^) equals 0, and I^2^ test. All the conducted meta-analyses evidenced the presence of significant heterogeneity, defined as a I^2^ > 55% and a Q statistic p value below 0.05. Accordingly, random effect models were applied. Finally, the likelihood of publication bias was estimated with a visual inspection of the funnel plot. All analyses were carried out using metaphor and meta-packages in R 3.6.1 software for Mac (R Foundation for Statistical Computing, Vienna, Austria).

## 3. Results

### 3.1. Study Selection

**Figure 1:**
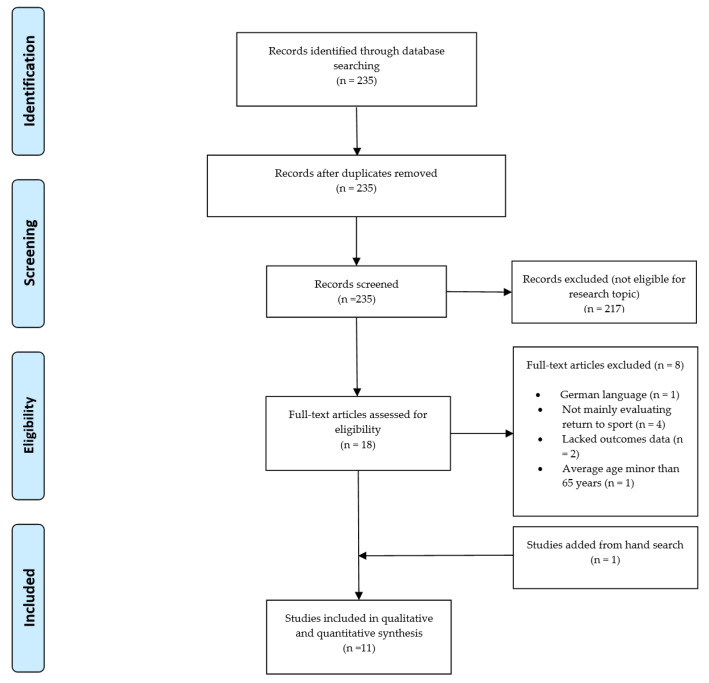
PRISMA flow-chart of included studies.

The initial database searches identified 235 potentially eligible papers. After reviewing title and abstract, 217 papers were excluded and 18 were selected for full-text evaluation. Out of these, eight papers were excluded for the following reasons: mean age of the cohort < 65 years (*n* = 1), German language (*n* = 1), return to sport not clearly stated (*n* = 4), and insufficient outcomes data (*n* = 2). One paper was added from hand search. At the end of the selection process, 11 studies were included in this systematic review and 11 papers were included in the meta-analysis [16,17,18,19,20,21,22,23,24,25]. The search process is summarized in the PRISMA flowchart (Figure 1) [14].

### 3.2. Study Characteristics and Demographic Details

Of the included studies, one was a single-center prospective cohort study (PCS) of level of evidence (LOE) III [19], four were retrospective studies (RS) of LOE III [18,20,23,25], one was a retrospective study of LOE IV [26], and five were case series of LOE IV [16,17,21,22,24]. All studies were published between 2010 [21] and 2018 [26]. The 11 included studies reported on 1254 shoulder arthroplasties in 1238 patients. Within the included studies, the number of subjects varied from 35 [24] to 276 [26]. The mean age of the cohorts was 72.5 years. The mean duration of follow-up was 3.7 years, ranging from 2.4 [26] to 6.2 years [17]. Four out of the 11 studies had a mean follow-up longer than four years [16,17,19,23]. The indications for surgery were several: rotator cuff arthropathy (522 patients), primary osteoarthritis (270 patients), fracture sequelae (37 patients), and rheumatoid arthritis (15 patients). Pre-operative diagnosis was not specified for 367 patients [19,22,23,26].

In total, 375 ATSA and 750 RTSA were implanted. The side of surgery was specified in 9 studies [16,17,18,19,20,21,22,23,25]. The dominant shoulder was involved in 544 patients, while the non-dominant shoulder was treated in 288 patients. Bilateral shoulder arthroplasty was performed in 17 patients [17,24]. The most frequent indication for ATSA was primary osteoarthritis without cuff disfunction [17,19,21,24]. Conversely, RTSA was performed in patients affected by primary osteoarthritis [16,20], cuff tear arthropathy [16,18,20,23,25], proximal humeral fractures [18,20,23], rheumatoid arthritis [18,20], and shoulder tumors [23]. Two studies did not specify surgical indications [22,26]. The main characteristics of the included papers are summarized in Table 1.

### 3.3. Methodological Evaluation

The MINORS score ranged from 7 [17,25] to 11 [23] for non-comparative studies and from 14 [16,18] to 16 [20,26] for the comparative ones (Table 1). The mean value was 8.5 for non-comparative studies and 14.5 for comparative studies. All papers resulted at high risk of bias.

### 3.4. Return to Sport

**Figure 2:**
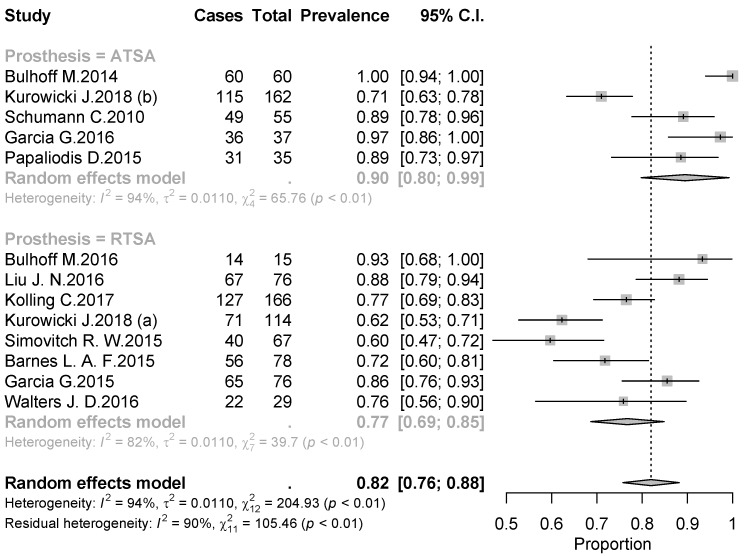
Forest plot chart of the combined rate of return to sports by meta-analysis with 95% confidence interval. ATSA, anatomic total shoulder arthroplasty; RTSA, reverse total shoulder arthroplasty.

**Figure 3:**
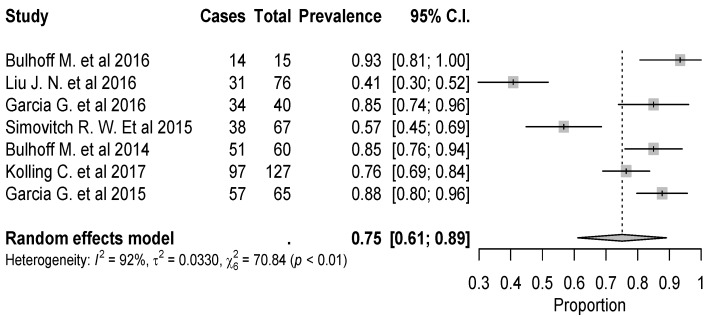
Forest plot chart of the rate of return to sports at the same or a higher level of play as before shoulder arthroplasty with 95% confidence interval.

**Figure 4:**
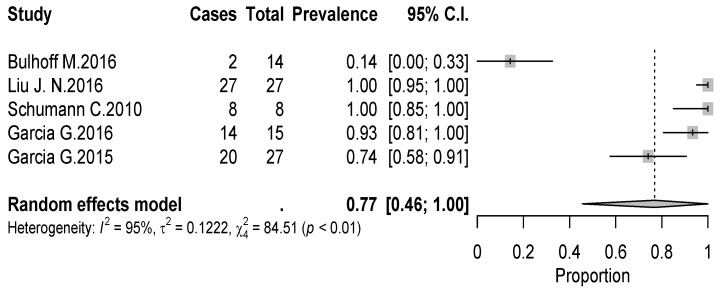
Forest plot chart of the rate of return to fitness after shoulder arthroplasty with 95% confidence interval.

**Figure 5:**
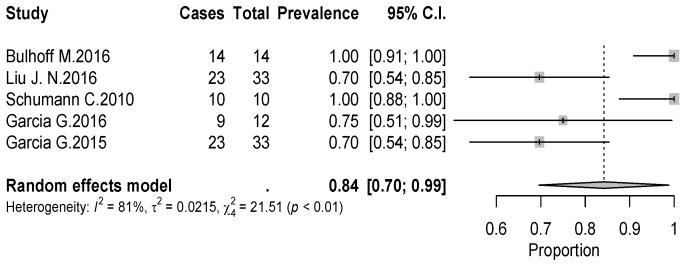
Forest plot chart of the rate of return to swimming after shoulder arthroplasty with 95% confidence interval.

**Figure 6:**
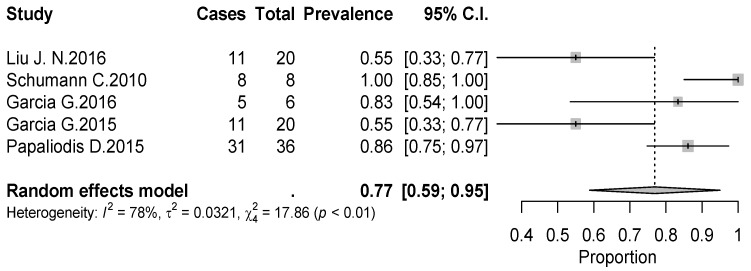
Forest plot chart of the rate of return to golf after shoulder arthroplasty with 95% confidence interval.

**Figure 7:**
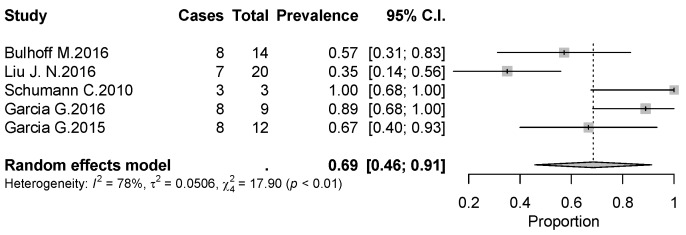
Forest plot chart of the rate of return to golf after shoulder arthroplasty with 95% confidence interval.

**Figure 8:**
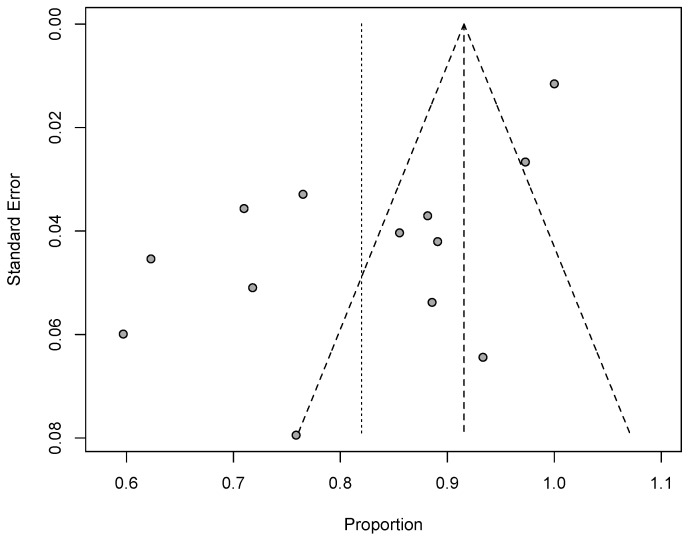
Funnel plot chart evaluating publication bias.

The overall rate of return to sport for elderly patients was 82% (95% CI 0.76–0.88, *p* < 0.01) (Figure 2). Patients undergoing ATSA returned at a higher rate (90%) (95% CI 0.80–0.99, *p* < 0.01) compared to RTSA (77%) (95% CI 0.69–0.85, *p* < 0.01) (Figure 2). The time to resume sports was reported in five studies [18,19,20,21,24] with a mean period of seven months ranging from 5.3 [18,20] to 11 months [15,21]. The results [16,17,18,19,20,25] showed that patients returned to sports activities at the same or a higher level in 75% of cases (95% CI 0.61–0.89, *p* < 0.01) (Figure 3). Six out of 11 studies [16,17,18,19,20,25] (54.5%) reported sport-specific rates of return. When combined by meta-analysis according to a random-effects model, swimming had the highest rate of return (84%), followed by fitness (77%), golf (77%), and tennis (69%) (Figure 4, Figure 5, Figure 6 and Figure 7). Regarding publication bias, the funnel chart was asymmetric, suggesting the presence of bias, particularly in smaller studies (Figure 8). The details of return to sport are reported in Table 2.

### 3.5. Clinical Outcome Data

**Figure 9:**
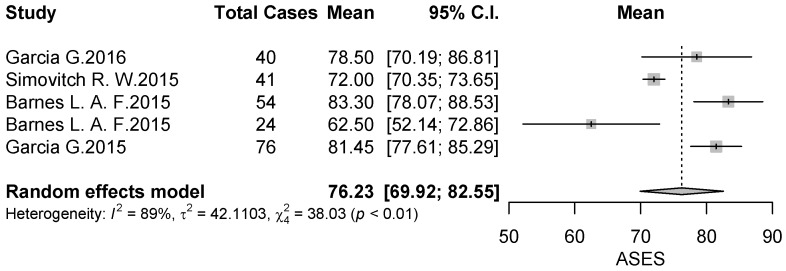
Forest plot chart of ASES score.

**Figure 10:**
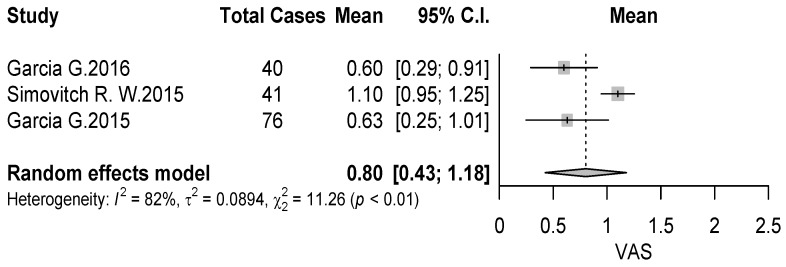
Forest plot chart of VAS score.

Outcome measures reported in the included studies are summarized in Table 3. The most frequently reported score was the American Shoulder and Elbow Surgeons (ASES) score, used in 6 (54%) of 11 studies [18,19,20,22,23,26], and the visual analog scale for pain (VAS) used in 4 (36%) studies [18,19,20,22] with an average of 76.23 (95% CI 0.81–0.90, *p* < 0.01) and 0.8 (95% CI 0.81–0.90, *p* < 0.01) points respectively (Figure 9 and Figure 10). Constant score was used in two studies [21,22] and the evaluation of range of motion (ROM) was used in two studies [22,23].

### 3.6. Rehabilitation Protocols

Only 5 out of 11 included papers reported the postoperative rehabilitation protocol [17,18,22,23,25]. Those authors advised a shoulder sling immobilization for the first four weeks, leaving free elbow and wrist movements. [17,18,22,23,25] In general, only passive ROM was allowed for the first 4 weeks, waiting for the sixth postoperative week to start active exercises. [17,18,22,23,25] Strengthening exercises were generally allowed from the twelfth postoperative week [18,22], even if Barnes et al. started them from the eighth [23]. On the contrary, Kolling et al. [25] permitted active mobilization and water therapy for shoulder strength and coordination from the second week after surgery. The surgical approach was evaluated in order to correlate subscapular repair to restrictions in the rehabilitative protocol. Among the five surgeons who performed the subscapularis tendon repair [16,17,21,23,25], only Kolling et al. [25] chose to limit external rotation movements to protect the reinserted tendon until the end of the second postoperative week. The postoperative rehabilitation protocols and the surgical approach are reported in Table 4.

## 4. Discussion

In the present review, we found that the overall rate of return to sport after ATSA and RTSA in elderly patients is 82%. Specifically, 90% of patients who underwent ATSA and 77% of patients who underwent RTSA were able to practice sports again. The fact that the pooled analysis demonstrated the highest rate of return to sports in ATSA is not unexpected. Several studies demonstrated greater range of motion, higher functional outcomes scores, and improved patient satisfaction when comparing ATSA and RTSA [27,28]. Among sports commonly performed after surgery, swimming has the highest rate (84%) followed by fitness (77%), golf (77%) and tennis (69%). Therefore, the most practiced sports after surgery are the non-contact ones, probably due to a defensive attitude of patients and surgeons. Golant et al. [29] have highlighted that, in the available literature, there is an extensive variation in surgeon recommendations on activity restrictions after TSA, and that information regarding return to sports activities after shoulder arthroplasty is also lacking. In particular, they find that surgeons recommend noncontact low-load sports at the expense of contact ones. Healy et al. [30] surveyed 35 members of the American Shoulder and Elbow Surgeons regarding their recommendations for sports participation after shoulder arthroplasty. They concluded that sports that may impart high loads on the glenohumeral joint, such as football, should be avoided, whereas low-impact sports, such as cross-country skiing and swimming, may be allowed.

Papaliodis et al. [24] demonstrated that return to sports is possible, reporting a significant decrease in shoulder pain during sports activity. In this study, all patients practiced golf. Thirty one of 35 patients could return to play golf after an average time of 8.4 months postoperatively (range, 2–24 months). Fifteen patients reported subjective improvement in their ability, 12 reported no change, and only 4 reported less ability. Schumann et al. [21] evaluated the return to sports activity after TSA in 55 patients. The most practiced sports were swimming (10 patients, 20.4%), golfing (8 patients, 16.3%), cycling (8 patients, 16.3%), and fitness (8 patients, 16.3%). Six patients did not resume sport activity after TSA. Of the considered patients, 33 of the 55 were able to resume sport within six months after surgery. Another 16 patients returned to practice sport within two years after TSA. In the study of Garcia et al. [20], 85.5% of patients resumed sports activity. Low contact sports and low demand sports had the highest rate of return to practice, (fitness: 81.5%, 22/27; swimming: 66.7%, 22/33; running 57.1%, 4/7; cycling 50.0%, 6/12; golf 50%, 10/20). Of the considered cohort, 47.6% resumed sport at a higher level than preoperative, while only 10.9% did not reach their preoperative activity level.

Moreover, the papers included in this systematic review confirmed a pain reduction after shoulder surgery. In the study of Liu et al. [18], the difference between preoperative and postoperative VAS was 5.64 points. In the study of Garcia et al. [20], the postoperative VAS score was 5.64 points lower than preoperative. Similar results were showed by Simovitch et al. [22], with a mean difference between preoperative and postoperative VAS of 6.1 points. Additionally, they showed that postoperative pain reduction was associated with an improvement of ROM and ASES scores [22]. Three studies reported the difference between preoperative and postoperative ASES and, in all of them, an improvement in postoperative values can be observed [18,20,22]. Barnes et al. [23] reported only the mean postoperative ASES which was 77.5, but even in this case, the improvement of ASES scores and VAS was associated with return to sport at the same or better preoperative level.

The present meta-analysis has shown that patients returned to sport activities at the same or a higher level in 75% of cases. This confirms that most patients undergoing shoulder arthroplasty (regardless of type) can safely return to at least one sport, with many returning to the same level of play, although a 100% guarantee should not be provided. Bulhoff et al. [17] assessed that, in their cohort, the postoperative activity levels and frequencies in sports practice were higher than before surgery. Moreover, patients were satisfied with their performances. Kolling et al. [25] selected 69 patients who clearly expressed their desire to resume sports activities after surgery and 60% of these patients were satisfied with their postoperative performance level and, within a year from surgery, 86% returned to practice sport at the same preoperative level or higher.

The postoperative rehabilitation protocol was reported in five studies [17,18,22,23,25]. Available protocols provided general information about time of immobilization and gradual recovery of shoulder motion and strength. Generally, the majority of surgeons followed similar indications: sling immobilization for at least four weeks, passive ROM for the first four weeks, active exercise from about the sixth postoperative week and strength training from the 12th postoperative week. On the contrary, Kolling et al. [25] permitted active mobilization and water therapy for shoulder strength and coordination in the second week after surgery. Unfortunately, the current literature lacks a detailed description of the rehabilitative steps and specific information about training for the athletic population. Moreover, to the best of our knowledge, no high-level evidence trials have been performed to test the efficacy of different post-operative rehabilitation protocols for patients who underwent TSA. However, some authors demonstrated that patients who received a physician-directed rehabilitation program had a significantly better range of motion as compared to patients only supervised by physiotherapists [12].

This systematic review has a few limitations including the number of studies and their heterogeneous methodological approach. Moreover, designs and implantation techniques may have varied significantly across the analyzed studies, thus reflecting the sparse available evidence on the subject and the absence of randomized controlled trials. Importantly, none of these studies mentions the abilities and experience of the surgeon. Since ATSA involves greater operative time and attention, surgical experience could be a determining factor in the decision to perform a reverse or anatomic total shoulder arthroplasty. In order to create a more homogenous cohort, future studies should account for these individual surgeon factors in the methodology. Moreover, all the included studies were affected by a high risk of bias and, in some of them, the follow-up period was quite short to detect important postoperative complications after return to sport, such as loosening or periprosthetic fractures. Patients and sports were heterogeneous as well as the postoperative rehabilitation protocol assessed. Great variability was observed in the postoperative treatment protocols following shoulder arthroplasty. Therefore, it is very difficult to identify common patterns, making it impossible to do a metanalysis of postoperative rehabilitation protocols. Moreover, we performed the metanalysis only on postoperative ASES and VAS scores since their preoperative data were not reported in the included studies, hindering the assessment of significant improvements of these postoperative outcomes. Finally, important postoperative clinical outcomes, such as postoperative ROM, were often not reported.

## 5. Conclusions

After ATSA and RTSA, elderly patients can satisfactorily resume their sports activities. The rate of return to sports following ATSA is slightly higher than RTSA, probably due to differences in the patient population, surgical indication, and biomechanical issues. Most patients are able to return to practice sport at the same or a higher preoperative level. The most practiced sports after surgery are low contact activities such as fitness, swimming, golf, and tennis. Unfortunately, there is a lack of research data on the advantages and disadvantages of existing rehabilitation protocols and no standard of practice could be deduced. Therefore, more prospective randomized studies are needed to establish which kind of postoperative protocol is best following ATSA and RTSA.

## Figures and Tables

**Table 1:** Study characteristics, demographic details and Methodological Index for Non-randomized Studies (MINORS) score.

Authors	Year of Publication	Study Design	LOE	N° of Shoulders (N° of Patients)	Dominant/Not Dominant	Mean Age (Years)	Type of Implant	Mean Follow-Up (Years)	MINORS Score
**Bulhoff et al. [17]**	2015	CS	IV	170 (154)	103/51	72	ATSA	6.2	7/16
**Bulhoff et al. [16]**	2016	CS	IV	38 (38)	29/9	Group A: 76.2; Group B: 78.4	RTSA	4.8	14/24
**Liu et al. [18]**	2016	RS	III	102 (102)	58/44	72.3	RTSA	2.6	14/24
**Kolling et al. [25]**	2017	RS	III	271 (271)	203/68	77.1	RTSA	2.9	7/16
**Schumann et al. [21]**	2010	CS	IV	100 (100)	60/40	66.2	ATSA	2.8	8/16
**Garcia et al. [19]**	2016	PS	III	40 (40)	26/14	66.3	ATSA	5.1	9/16
**Papaliodis et al. [24]**	2015	CS	IV	36 (35)	NR	67.2	ATSA	3.2	8/16
**Simovitch et al. [22]**	2015	CS	IV	67 (67)	NR	73	RTSA	3.6	10/16
**Barnes et al. [23]**	2015	RS	III	78 (78)	48/30	75.3	RTSA	4.8	11/16
**Garcia et al. [20]**	2015	RS	III	76 (76)	46/30	74.8	RTSA	2.6	16/24
**Kurowicki et al. [26]**	2018	RS	IV	276 (276)	NR	RTSA: 75 ATSA: 69	RTSA ATSA	2.4	14/24

ATSA: anatomic shoulder arthroplasty; RTSA: reverse total shoulder arthroplasty; CS: case series; RS: retrospective study; PS: prospective study; LOE: level of evidence; NR: not reported. Group A: patients who practiced sports within the last 5 years prior to shoulder replacement surgery; Group B: patients that have never participated in sports activities.

**Table 2:** Return to sport.

Study	Sports	Number of Patient Practicing Sport (%)	Number of Evaluated Patients	Rate of Return to Sport for Single Sport (%)	Overall Rate of Return to Sport (%)
**Schumann et al. [21]**	Swimming	10 (20.4%)	55	NR	49 (89%)
	Golf	8 (16.3%)			
	Cycling	8 (16.3%)			
	Fitness	8 (16.3%)			
	Other	21 (30.7%)			
**Papaliodis et al. [24]**	Golf	35 (100%)	35	NR	31 (88.57%)
**Garcia et al., 2015 [20]**	Fitness	27	76 (some patients practiced more than 1 sport)	22 (81.5%)	65 (85.5%)
	Swimming	33		22 (66.7%)	
	Golf	20		10 (50%)	
	Cycling	10		5 (50%)	
**Bulhoff et al., 2015 [17]**	Swimming	60 (57%)	105	60 (57%)	60 (100%)
	Fitness including lower limb			42 (27%)	
	Skiing			31 (30%)	
	Gardening			29 (28%)	
	Bowling/skittles			18 (17%)	
	Tennis			15 (14%)	
	Handball			6 (4%)	
	Athletics			4 (3%)	
	Volleyball			3 (2%)	
	Golf			2 (1%)	
	Other			26 (25%)	
**Bulhoff et al., 2016 [16]**	Swimming	15 (71%)	22	14 (100%)	14 (93%)
	Fitness including lower limb			8 (57%)	
	Skiing			7 (50%)	
	Gardening			8 (57%)	
	Bowling			7 (50%)	
	Tennis			8 (57%)	
	Handball			2 (14%)	
**Kolling et al. [25]**	Calisthenics	166	305	28 (17%)	127 (77%)
	Hiking			28 (17%)	
	Swimming			26 (16%)	
	Alpine skiing			13 (8%)	
	Tennis			12 (7%)	
	Others			58 (35%)	
**Liu et al. [18]**	Single tennis	12 (12.2%)	102	4 (33%)	87 (85.9%)
	Double tennis	18 (18.3%)		3 (16.6%)	
	Baseball	1 (1.02%)		1 (100%)	
	Swimming	33 (33.66%)		23 (70%)	
	Fitness	27 (27.54%)		27 (100%)	
	Golf	20 (20.4%)		11 (55%)	
	Cycling	12 (12.2%)		8 (67%)	
	Fishing	4 (4.8%)		1 (25%)	
	Rowing	1 (1.02%)		1 (100%)	
	Running	7 (7.14%)		5 (71.4%)	
	Skiing	7 (7.14%)		2 (29%)	
	Dancing	2 (2.04%)		1 (50%)	
	Horseback riding	2 (2.04%)		1 (50%)	
	Basketball	1 (1.02%)		1 (100%)	
**Simovitch et al. [22]**	Golf	67 (26%)	255	50 (75%)	64 (95%)
	Swimming			19 (29%)	
	Water aerobics			16 (24%)	
	Deep sea fishing			14 (21%)	
	Firearm sports			14 (21%)	
	Weight lifting			12 (18%)	
	Softball			7 (11%)	
	Tennis			7 (11%)	
	Table tennis			5 (7%)	
	Scuba diving			5 (7%)	
	Racquetball			3 (5%)	
	Surfing			1 (2%)	
	Water skiing			1 (2%)	
**Garcia 2016 et al. [19]**	Golf	6 (8.3%)	72	5 (83.3%)	65 (90.27%)
	Swimming	12 (16%)		9 (75%)	
	Baseball	1 (1.4%)		1 (100%)	
	Basketball	1 (1.4%)		1 (100%)	
	Nature sports	7 (9.7%)		7 (100%)	
	Fitness	15 (21%)		14 (93%)	
	Single tennis	5 (7%)		4 (80%)	
	Running	14 (19.4%)		13 (92.9%)	
	Cycling	5 (7%)		5 (100%)	
	Softball	2 (2.7%)		2 (100%)	
	Double tennis	4 (5.5%)		4 (100%)	
**Barnes et al. [23]**	High intensity activities (hunting, golf, skiing …)	NR	78	18 (23.1%)	100%
	Moderate intensity activities (swimming, bowling …)			38 (48.7%)	
	Low intensity activities (riding bike, walking, dancing …)			22 (28.2%)	
**Kirowicki et al. [26]**	Golf	RTSA: 16 (22%) ATSA: 31 (27%)	RTSA: 71 ATSA 115	NR	RTSA 71/114 (62%); ATSA 115/162 (70%)
	Swimming	RTSA: 13 (18%) ATSA: 19 (16%)			
	Walking	RTSA: 16 (22%) ATSA: 18 (15%)			
	Gym exercises	RTSA: 8 (11%) ATSA: 24 (20%)			
	Racquet sport	RTSA: 4 (5%) ATSA: 13 (11%)			
	Group fitness	RTSA: 5 (7%) ATSA: 10 (8%)			
	Fishing and target shooting	RTSA: 5 (7%) ATSA: 4 (3%)			
	Adventure sport	RTSA: 1 (1%) ATSA: 9 (8%)			

NR: not reported.

**Table 3:** Clinical outcome data of the included studies.

Authors	Implant	Outcomes		Complication (Number)	Main Conclusion
		Preoperative	Postoperative		
**Bulhoff et al. [17]**	ATSA	NR		NR	- Patients with active sports participation before TSA are successfully able to return to sports activities after surgery. - Patients who are not participating in sports just before surgery are unlikely to resume sports after surgery.
**Bulhoff et al. [16]**	RTSA	NR		aseptic loosening of glenoid component (1), dislocation (2)	Patients with glenohumeral osteoarthritis and rotator cuff disease being active prior to RSA surgery are able to successfully return to their level of sports participation afterwards.
**Liu et al. [18]**	RTSA	**ASES SCORE (overall mean change)** +39		None	Despite traditional sport restrictions placed on RTSA, patients undergoing RTSA can return to sports at rates higher than those undergoing HHA, with fewer postoperative complaints.
		**VAS (overall mean reduction)** −5.64			
**Kolling et al. [25]**	RTSA	NR		NR	- Most patients carried out their main sports activity after surgery with a moderate level of intensity (83%) and between one to three times per week (69%). - 42% indicated that returning to sports was among their key demands after RSA.
**Schumann et al. [21]**	ATSA	**CONSTANT SCORE (mean ± SD)**		NR	- The probability of being able to do sports postoperatively—if done preoperatively—is high. - Long-term studies are needed to determine whether the greater loading on the joint will lead to more rapid wear and a higher rate of loosening with time.
		NR	GI: 70.8 ± 13.8; GII: 77.2 ± 10.6; GIII: 69.3 ± 9.7		
		**SF-36 (mean ± SD)**			
		NR	Physical component: GI 41.0 ± 11.2; GII: 46.2 ± 9.0; GIII: 42.2 ± 10.6; Mental component GI: 55.6 ± 9.3; GII 55.7 ± 6.4; GIII: 47.7 ± 12.9		
		**DASH SCORE (mean ± SD)**			
		NR	GI: 76.6 ± 19.3; GII: 83.4 ± 12.7; GIII: 69.6 ± 18.6		
		**SPADI SCORE (mean ± SD)**			
		NR	GI: 78.6 ± 20.5; GII: 83.7 ± 16.5; GIII: 68.7 ± 19.2		
**Garcia et al. [19]**	ATSA	**ASES SCORE (mean)**		NR	- Rate of return to sports was significantly better after TSA, although further studies are needed to review glenoid loosening. - HA patients had significantly more pain, worse satisfaction, and a decreased ability to return to sports.
		34.0	78.5		
		**VAS (mean)**			
		6.1	0.6		
**Papaliodis et al. [24]**	ATSA	**VAS (mean average improvement)** 4.3		NR	- Patients who undergo TSA for primary glenohumeral arthritis can safely return to golfing activity with a significant decrease in their perceived pain level. - Statistically significant findings included an increase in driving distance by 12.5 yd and an improvement in handicap by 1.4.
**Simovitch et al. [22]**	RTSA	**CONSTANT SCORE (mean ± SD)**		type II acromion stress fracture (1); postoperative infection (1), postoperative dislocation (1)	- RTSA in senior athletes can be safely performed with good clinical results. - No prominent mode of mechanical or clinical failure has been identified with short-term follow-up.
		25 ± 1.9	84 ± 1.7		
		**ASES SCORE (mean ± SD)**			
		31 ± 1.9	72 ± 4.5		
		**ROM (mean ± SD)**			
		Flexion: 78 ± 16; Abduction: 67 ± 14.6; External rotation: 26 ± 5.2	Flexion: 152 ± 12; Abduction: 148 ± 11.6; External rotation: 44 ± 5.7		
		**VAS (mean ± SD)**			
		7.2 ± 0.5	1.1 ± 0.5		
		**SSV (mean ± SD)**			
		27 ± 4.3	90 ± 4		
**Barnes et al. [23]**	RTSA	**ASES SCORE (mean)**		dislocation (3), aseptic loosening (1); dissociated glenosphere baseplates (1); deep infections (2); superficial infection (1)	RTSA results in good pain relief and motion, with a variety of postoperative overhead activities enjoyed by some patients who are not limited by comorbidities.
		NR	77.5		
		**ROM (mean)**			
		NR	active forward elevation: 140°, active external rotation: 48°, active internal rotation: S1		
		**VAS (mean)**			
		NR	2.3		
**Kurowicki et al. [26]**	RTSA ATSA	**ASES SCORE (mean)**		NR	- Both TSA and RSA allow for participation in work and sports, with TSA patients reporting better overall ability to participate. - For sports involving shoulder function, TSA patients more commonly report maximal ability to participate than RSA patients.
		NR	RTSA: 77.14 ATSA: 83.03		
**Garcia et al. [20]**	RTSA	**ASES SCORE (mean ± SD)**		None	- Patients undergoing RTSA had an 85% rate of return to 1 or more sporting activities at an average of 5.3 months after surgery. - Noncontact, high-demand activities (swimming, skiing, golf, and tennis) had lower return rates than lower demand activities. - Age greater than 70 years old was a significant predictor of decreased return to activities.
		34.3 ± 17.2	81.45 ± 17.1		
		**VAS (mean ± SD)**			
		6.57 ± 2.4	0.63 ± 1.7		

ATSA: anatomic shoulder arthroplasty; RTSA: reverse total shoulder arthroplasty; NR: not reported; ASES score: American Shoulder and Elbow Surgeons Score; VAS: visual analogue scale; SF-36: Short For,-36; DASH score: Disabilities of the Arm, Shoulder and Hand Score; SPADI score: Shoulder Pain and Disability Index Score; ROM: range of motion; SSV: subjective shoulder value; SD: standard deviation.

**Table 4:** Rehabilitative protocols and surgical approach.

Authors	Rehabilitative Protocols	Surgical Approach
**Bulhoff et al. [17]**	1. Abduction pillow (20°) and internal rotation (20°) for the first 4 weeks. 2. Day 1 to 6th week: daily actively assisted exercise with a physiotherapist. 3. From 6th week: active and free range of motion.	Deltopectoral approach with subscapularis repair
**Bulhoff et al. [16]**	NR	Deltopectoral approach with subscapularis repair
**Liu et al. [18]**	1. Sling immobilization for the first 4 weeks. 2. From 2nd week: passive ROM at 2 weeks. 3. From 6th week: active ROM. 4. From 12th week: strengthening exercises and prior recreational activities and work were encouraged. Restriction: avoid contact sports	NR
**Kolling et al. [25]**	1. Sling immobilization during the night for the first 4 weeks. 2. From day 1 to 2nd week: passive motion with only limited external rotation movements to protect the reinserted subscapularis tendon. 3. From 2nd to 5th week: active mobilization and water therapy to gain shoulder strength and coordination. 4. After 12th week: resume any prior sports activities including non-contact sports.	Deltopectoral approach with subscapularis repair
**Schumann et al. [21]**	NR	Deltopectoral approach with subscapularis repair
**Garcia et al. [19]**	NR	Deltopectoral approach
**Papaliodis et al. [24]**	NR	NR
**Simovitch et al. [22]**	1. Abduction sling for the first 4 weeks. 2. From day 1 to 4th week: passive ROM and isometric exercises. 3. From 6th week: active ROM. 4. From 12th week: strengthening exercises. 5. From 16th week: return to sports.	Deltopectoral approach without subscapularis repair
**Barnes et al. [23]**	1. Sling immobilization for the first 4 weeks (only wrist and elbow motion allowed) 2. From 4th week: active shoulder ROM. 3. From 8th week: strengthening exercises.	Deltopectoral approach with subscapularis repair
**Garcia et al. [20]**	NR Restriction: avoid contact sports.	NR
**Kirowicki et al. [26]**	NR	NR

NR: not reported; ROM: range of motion.
